# Is the effect of melatonin on vascular endothelial growth factor receptor-2 associated with angiogenesis in the rat ovary?

**DOI:** 10.6061/clinics/2019/e658

**Published:** 2019-02-27

**Authors:** Yasemin Behram Kandemir, Esma Konuk, Ertan Katırcı, Feride Xxx, Mustafa Behram

**Affiliations:** I Harran University, Faculty of Medicine, Department of Anatomy, Şanlıurfa, Turkey.; II Akdeniz University, Faculty of Medicine, Department of Histology, Antalya, Turkey.; III Kanuni Sultan Süleyman Hospital, Department of Perinatology, Istanbul, Turkey.

**Keywords:** Melatonin, Ovary, VEGF, VEGFR1, VEGFR2

## Abstract

**OBJECTIVES:**

Vascular endothelial growth factor (VEGF) and its receptors play important roles in angiogenesis. Melatonin plays an important role in gonadal development; thus, its effect on the reproductive system is evident. We investigated the influence of melatonin on the expression of VEGF, vascular endothelial growth factor receptor-1 (VEGFR1) and vascular endothelial growth factor receptor-2 (VEGFR2), as well as on changes in oxidative stress markers and follicle numbers in rat ovaries.

**METHODS:**

For this purpose, 45 Wistar rats were separated into the following groups: Group 1, control; Group 2, vehicle; and Group 3, melatonin. Rats in Group 3 were treated with melatonin at 50 mg/kg/day for 30 days. The effects of melatonin on the expression of VEGF, VEGFR1 and VEGFR2 were established by immunohistochemistry analysis. The effects of melatonin on antioxidant enzyme activities were demonstrated by spectrophotometric analysis.

**RESULTS:**

Based on immunohistochemistry analysis, VEGFR2 was predominantly localized to theca cells in the ovary. Our data indicate that melatonin treatment can significantly increase VEGF and VEGFR1 expression in the ovary ( *p* <0.05). Additionally, the number of degenerated follicles significantly decreased with melatonin treatment ( *p* <0.05). Melatonin administration also led to significant increases in antioxidant enzyme levels in the ovary.

**CONCLUSION:**

Melatonin treatment exerts protective effects on follicles against increased lipid peroxidation through modulating tissue antioxidant enzyme levels. These effects may be related to angiogenesis and antioxidant activities.

## INTRODUCTION

Vascular endothelial growth factor (VEGF) and its receptors play fundamental roles in complex physiological processes, such as angiogenesis by which new blood vessels develop from existing vessels ( 1 ).

Melatonin (N-acetyl-5-methoxytryptamine) is a lipophilic hormone with various physiological roles in mammals; it is synthesized from the amino acid tryptophan and is released from pinealocytes in the pineal gland during periods of darkness ( 2 ). Melatonin, a powerful antioxidant and free radical scavenger, supports the maturation and ovulation of follicles and protects follicles from oxidative stress, thus exerting a positive effect on reproductive functions ( 3 , 4 ). Melatonin is closely related to sex steroids, especially in the reproductive system ( 5 , 6 ). Melatonin alters sex hormone synthesis by inhibiting steroidogenesis via direct changes in the cAMP levels of theca cells ( 7 ). Importantly, melatonin, which exists in the ovary, impacts reproductive functions ( 8 , 9 ). Many studies have emphasized that melatonin has multiple protective effects on pathological and physiological conditions ( 10 ). Endothelial cell migration is an essential step in the process of angiogenesis, and the migration of endothelial cells is initiated by luteinizing hormone secretion in the ovarian follicle ( 11 ). In mammals, the angiogenesis of luteinized follicles is increased by luteinizing hormone secretion ( 12 ). In one study, the administration of exogenous melatonin was effective at increasing the concentrations of gonadotropins, such as follicle stimulating hormone and luteinizing hormone ( 13 ). Notably, follicle stimulating hormone and luteinizing hormone are related to the synthesis of growth factors. VEGF, one of these growth factors, is closely related to angiogenesis in the follicular phase. VEGF is important in the nutritional support and development of the corpus luteum and stroma ( 14 ). Some studies have focused on the immunoreactivity of VEGF in the ovary ( 15 , 16 ). However, there is minimal information available on the role of melatonin in VEGF, VEGFR1, and VEGFR2 expression in vital biological functions such as angiogenesis and reproduction. This study focuses on a similar paradigm, including melatonin treatment, VEGF expression, and antioxidant enzyme activity. Therefore, the present research examines the effects of melatonin on VEGFA and its receptors (VEGFR 1 and VEGFR2), as well as the activity of antioxidant enzyme levels and lipid peroxidation in adult female rat ovaries.

## MATERIALS AND METHODS

### Animals

This study was conducted on 45 female Wistar rats aged between 8 and 10 weeks and weighing 350–450 g. In this study, which was planned in accordance with the standards determined by the Institutional Animal Care and Use Committee at Akdeniz University Medical School (Animal Committee Review and Approval No: 2018.01.014), animals were divided into the following groups: Group 1, control (C, n=15); Group 2, vehicle (V, n=15); and Group 3, melatonin (M, n=15) ( Table 1 ). The melatonin dose and administration protocol were based on our previous studies ( 17 , 19 ). Melatonin was administered by intraperitoneal (i.p.) injection at 50 mg/kg/day for 30 days in the M group; the same volume of 10% ethanol (used as a solvent for melatonin) was administered to the V group.

**Table 1:** - Animal groups.

Groups	Melatonin-treated	Injected	n
Group l: Control (C)		-	15
Group 2: Vehicle (V)		10% Ethanol	15
Group 3: Melatonin (M)		50 mg/kg/day Melatonin	15

### Histologic analysis

Dissected ovaries were placed in formaldehyde for 12h for histological analysis. Ovaries were cryosectioned at 5 μm using a cryostat. The sections were stained with haematoxylin-eosin and analysed under a light microscope.

### Immunohistochemical staining

Immunohistochemical analysis was performed according to a well-established method ( 20 ). Frozen ovarian tissue sections were air-dried for 30 min at room temperature. These sections were washed with PBS twice for 5 min each. The sections were treated with 3% hydrogen peroxidase in methanol to quench endogenous peroxidase activity and subsequently washed with PBS. Ovarian tissue sections were incubated with a blocking solution for 7 min at room temperature in a humidified chamber. These sections were incubated with VEGF (Abcam, ab46154, dilution; 1/50), VEGFR1 (Abcam, ab2350, dilution; 1/25) and VEGFR2 (Abcam, ab39256, dilution; 1/50) antibodies at +4°C overnight. The following day, the sections were incubated with SignalStain Boost IHC Detection Reagent (Cell Signaling, 8114). The reaction products were visualized using Dab (Cell Signaling, #8059). These sections were counterstained with Mayer’s haematoxylin and mounted with Entellan. Then, images were taken with a light microscope.

### Immunofluorescence staining

As previously described, immunofluorescence analyses were performed ( 19 ). Frozen ovarian tissue sections were air-dried for 30 min at room temperature. These sections were washed with PBS twice for 5 min each and incubated with 2.5% normal goat serum (Vector, S-1012) for 1h at room temperature in a humidified chamber. Subsequently, these sections were incubated overnight at +4°C with VEGF (Abcam, ab46154, dilution; 1/100), VEGFR1 (Abcam, ab2350, dilution; 1/100) and VEGFR2 (Abcam, ab39256, dilution; 1/500) antibodies. The next day, the sections were incubated with secondary antibodies for 45 min in darkness.

### Fluorescence microscopy

Immunoreactive staining measurements were performed as described in our previous study ( 21 ). A Zeiss Stemi SV11 stereomicroscope was used to measure fluorescence intensity. Fluorescent images acquired via a rhodamine filter were compared utilizing the 8 BPP greyscale format whereby each pixel contains 8 bits of information codifying brightness, with a range of 0 to 250. The scale for pixel brightness or the pixel grey value was constructed such that higher numbers indicate greater pixel brightness. Digital images were captured with a slow scan CCD camera (Spot RT, Diagnostic Instruments, Scientific Instrument Company, Inc., Campbell, CA, USA). For the quantification of pixel brightness, images were captured using a ×25 objective and Image-Pro Plus Software Version 6.2 (Media Cybernetics Rockville, MD, USA). The exposure time was optimized to ensure that only a few pixels were saturated at 250 grey values. However, all images representing the same labelling were taken under the same exposure conditions. An interactive threshold was used to detect the pixel brightness of minimum fluorescence. Threshold values ensured the inclusion of the entire signal range in the sample. This value was further used to extract and compare the pixel number between animals of the same group and between experimental groups.

### Biochemical analyses

Ovary samples were sonicated (BandelinSonopuls, HD 2070, Bandelin Electronic GmbH & Co. KG, Berlin, Germany) in 500 μl ice-cold buffer (50 mM potassium phosphate pH 7.0, 1 mM EDTA) and centrifuged (thiobarbituric acid reactive substance (TBARS); 15,000 g for 10 min at 4°C, glutathione peroxidase (GPx); catalase (CAT); 10,000 g for 15 min at 4°C, superoxide dismutase (SOD); 1,500 g for 5 min at 4°C). The supernatants were collected and stored at -80°C for later biochemical analysis according to well-established biochemical analysis methods ( 18 ).

### Measurement of superoxide dismutase (SOD) activity

SOD activity was assessed using an SOD activity assay kit (Cayman Chemical, Ann Arbor, USA) in accordance with the previously methods described by Misra and Fridovich, Kaya et al. ( 18 , 22 ). For the evaluation of SOD activity, the xanthine oxidase-hypoxanthine system, which continuously forms the superoxide anion, was used.

### Measurement of catalase (CAT) activity

An assay kit (Cayman-707002) and spectrophotometric analysis were used to measure CAT enzymatic activity in ovary tissues in accordance with the methods previously described by Aebi, Kaya et al. ( 18 , 23 ) and were expressed in units per milligram of protein at 25°C.

### Measurement of glutathione peroxidase (GPx) activity

Glutathione peroxidase activity was determined indirectly by the coupled reaction with glutathione reductase using a GPx assay kit (Sigma–Aldrich Chemie, Steinheim, Germany) in accordance with the methods previously described by Paglia and Valentine, Kaya et al. ( 18 , 24 ). Oxidized glutathione was converted to the reduced state by glutathione reductase, which was accompanied by the oxidation of NADPH to NADP with a decrease in absorbance of 340 nm. One unit of the enzyme that causes the oxidation of NADPH per min at 25°C is defined as an enzyme activity unit, as we previously described ( 18 ).

### Thiobarbituric acid reactive substance (TBARS) assay

As described in earlier studies ( 18 , 25 ), using a fluorometric method, we determined ovary TBARS levels (MDA; malondialdehyde) using 1,1,3,3-tetraethoxypropane as a standard. The protein concentrations were analysed spectrophotometrically according to a modified Bradford method using bovine serine albumin as the standard (Shimadzu RF-5500, Kyoto, Japan), as we previously described ( 18 ).

### Statistical analysis

One-way ANOVA with post hoc Tukey’s test was applied for statistical analyses, and significance levels were determined as 0.05 (Statistica 6.0 software; Stat Soft, Tulsa, OK, USA).

## RESULTS

### Antioxidant enzyme activities and lipid peroxidation in ovarian tissues

**Figure 1:**
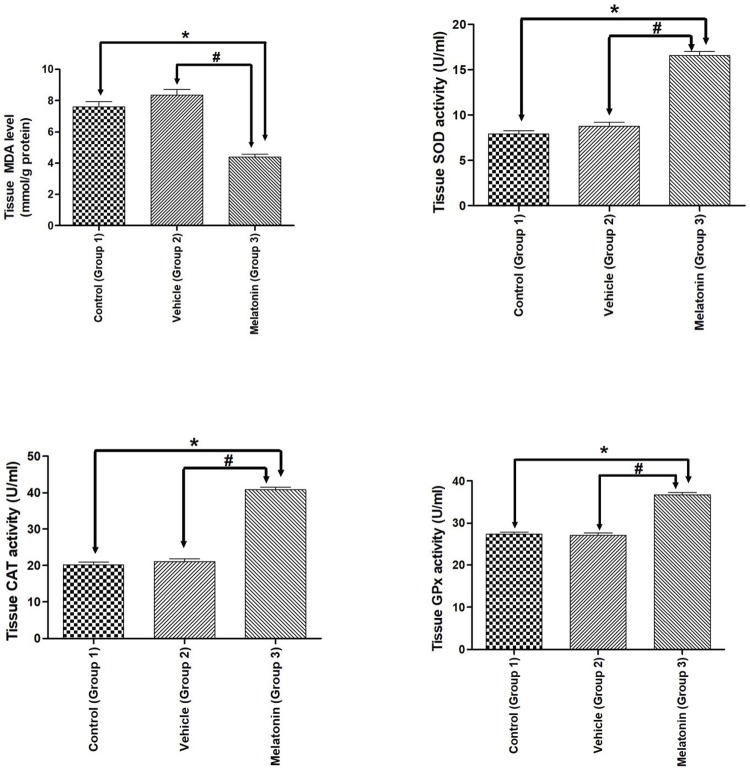
- Antioxidant enzymes and MDA levels.

MDA, SOD, CAT, and GPx enzyme activities established for the studied groups are summarized in Figure 1 . A beneficial effect of melatonin treatment was found by comparing SOD, CAT, GPx activities and MDA levels in ovarian tissues. Compared to vehicle treatment, melatonin treatment significantly increased all antioxidant enzyme activities; however, melatonin administration significantly attenuated MDA levels in the melatonin group ( *p* <0.05). There was no significant difference between the control and vehicle groups ( Figure 1 ).

Diagram showing the antioxidant enzymes and MDA levels of the animal groups. Note that MDA levels were significantly reduced, while antioxidant enzymes were significantly increased after melatonin administration in both experimental paradigms. Data are shown as the mean ± S.E.M.; n = 15 rat/group. *, *p* <0.05 indicates the significance compared to the respective control values, #, *p* <0.05 indicates the significance compared to the respective vehicle values.

### Effect of melatonin treatment on healthy and degenerated follicles

**Figure 2:**
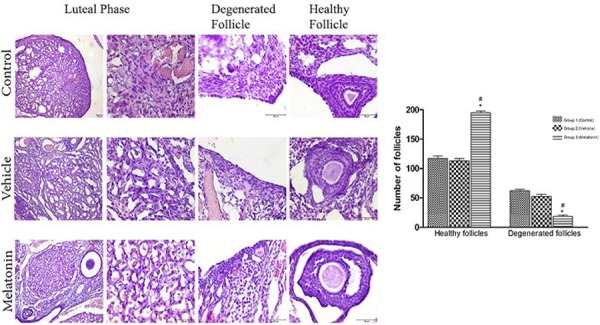
- Histomorphometric analysis.

We analysed the association between the number of follicles (healthy and degenerated) and melatonin treatment, which showed that melatonin treatment may affect the number of follicles in the rat ovary. Compared with non-melatonin treatment, melatonin treatment significantly increased the number of healthy follicles. In contrast, melatonin treatment was associated with a marked decrease in the number of degenerated follicles ( Figure 2 ).

Histomorphometric assessment of healthy and degenerated follicles in the rat ovary. Scale bars represent 50 μm. Comparison of the number of healthy and degenerated follicles among the three groups. Data are shown as the mean ± S.E.M.; n = 15 rat/group. *, *p* <0.05 indicates significance compared to the respective vehicle values. #, *p* <0.05 indicates significance compared to the respective control values.

### Immunohistochemistry and immunofluorescence analyses

**Figure 3:**
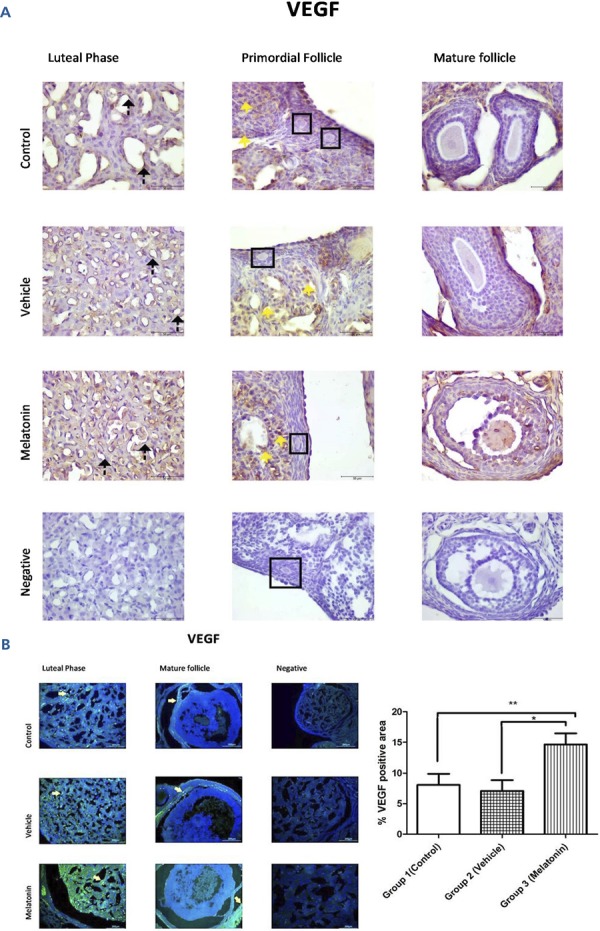
Immunoreactivity of VEGF proteins.

**Figure 4:**
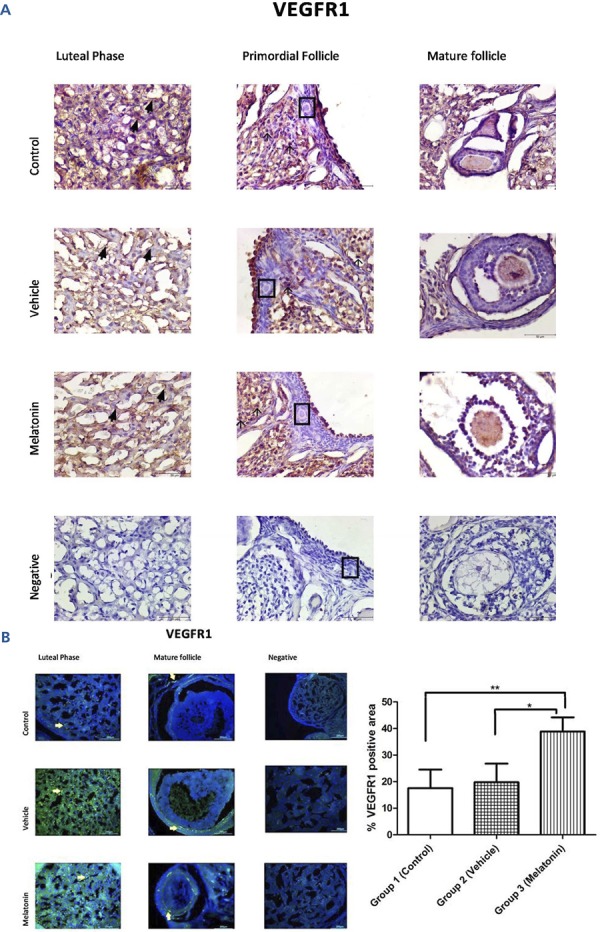
Immunoreactivity of VEGFR1 proteins.

**Figure 5:**
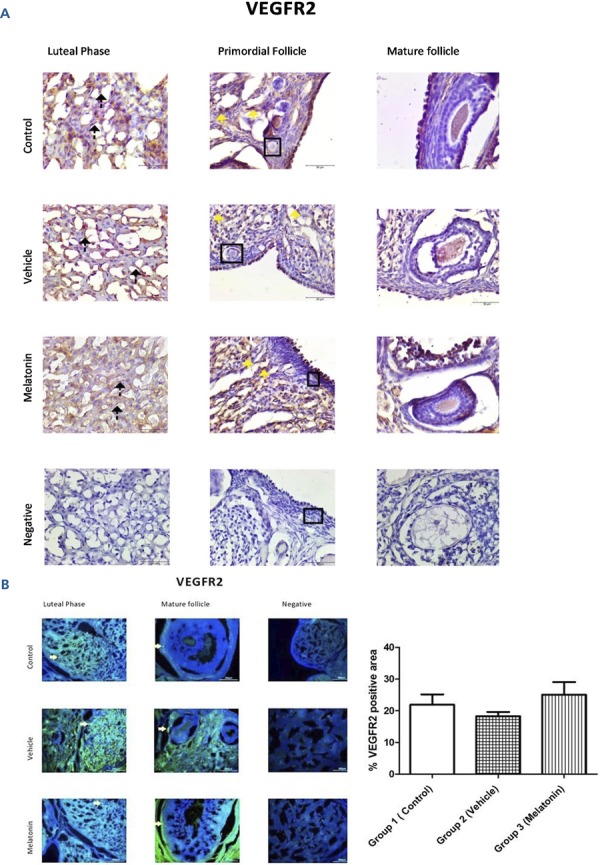
Immunoreactivity of VEGFR2 proteins.

The immunohistochemistry and immunofluorescence results showed that VEGF ( Figure 3 ), VEGFR1 ( Figure 4 ), and VEGFR2 ( Figure 5 ) were expressed in stromal cells and endothelial cells. In particular, VEGFR2 was expressed in theca cells where vascularity is greater in active follicles. There was a significant difference in the immunoreactivity of VEGF and VEGFR1 between the control and melatonin groups. Conversely, VEGF, VEGFR1, and VEGFR2 were not expressed in the granulosa cells of primordial follicles. In our experiments, although high levels of VEGF and VEGFR1 proteins were detected in the melatonin group, there was no significant difference between the control and vehicle groups. Immunofluorescence revealed positive staining for both VEGF and its receptors (VEGFR1 and VEGFR2) in blood vessels and the active follicle. The immunoreactivity of VEGF and VEGFR1 was higher in the melatonin group than in the control group. Our preliminary results have the potential to inform future research in this field.

A: Immunohistochemical staining, B: Immunofluorescence staining

Immunoreactivity of VEGF proteins in Group 1 (control), Group 2 (vehicle), and Group 3 (melatonin). An increase in VEGF in the endothelial cells of the blood vessel (as indicated by yellow arrows) can be seen in the melatonin treatment group. Data are displayed as the mean ± S.E.M.; n=15 rats/group. **, *p* <0.05 indicates significance compared to the respective control values. *, *p* <0.05 indicates significance compared to the respective vehicle values.

A: Immunohistochemical staining, B: Immunofluorescence staining.

Immunoreactivity of VEGFR1 proteins in Group 1 (control), Group 2 (vehicle), and Group 3 (melatonin). An increase in the immunoreactivity of VEGFR1 in theca cells (as indicated by yellow arrows) can be seen in the melatonin treatment group. Data are displayed as the mean ± S.E.M.; n=15 rats/group.**, *p* <0.05 indicates significance compared to the respective control values. *, *p* <0.05 indicates significance compared to the respective vehicle values.

A: Immunohistochemical staining, B: Immunofluorescence staining.

Immunoreactivity of VEGFR2 proteins in Group 1 (control), Group 2 (vehicle), and Group 3 (melatonin). Immunoreactivity of VEGFR2 in granulosa cells as indicated by yellow arrows can be seen in all of the groups. Data are displayed as the mean ± S.E.M.; n=15 rats/group.

## DISCUSSION

The cell biochemical pathways underlying the angiogenesis of the ovary are not fully understood. Understanding these mechanisms is critical because the fate of follicles in terms of whether they undergo ovulation or atresia is determined during this process ( 26 ). According to different studies, VEGF is a factor in angiogenesis as it promotes angiogenesis by activating endothelial cell proliferation and migration ( 27 , 28 ). Moreover, VEGF secretion is increased by melatonin treatment in various tissues ( 29 ). Melatonin administration may affect steroidogenesis in the ovary ( 30 , 31 ). There is supporting evidence for the mechanism of melatonin’s effect on ovarian tissue, even in ovarian tumour cells, indicating that melatonin regulates the secretion of VEGF. Melatonin can bind MT1 receptors to reduce VEGFR2 and hypoxia-inducible factor (HIF)-1α in ovarian tumour cells; this finding is in contrast to the results observed in this study with normal cells ( 32 - 34 ). Melatonin is a potent antioxidant and free radical scavenger that promotes ovarian cell survival, decreases atresia and develops in vitro fertilization rates and oocyte quality ( 9 , 35 , 36 ). Many reports have suggested that the ovary is capable of producing melatonin at different seasonal levels, while others have emphasized the link between melatonin and follicle quality as well as ovarian function ( 19 , 37 , 38 ). Melatonin also increases VEGF, a protein that controls angiogenesis, and VEGF receptors are expressed in the pituitary gland and are under the control of melatonin secretion ( 39 ). The data from the present study are supported by the literature, showing that melatonin treatment significantly increases the elevated immunoreactivity of VEGF and VEGFR1, particularly in areas where vasculogenesis is high.

Melatonin is a crucial antioxidant that increases the oxidative stress allowance by activating antioxidant enzymes such as SOD, CAT, and GPx while simultaneously scavenging ROS ( 40 ). Oxidative stress occurs when free radicals formed in the body exceed the number of free radicals released from the body. The indicator of this disturbed balance is the MDA level, resulting from lipid peroxidation. Our findings are in line with the literature in that melatonin treatment significantly decreased MDA levels in rat ovaries ( *p* <0.05) ( 41 , 42 ). Moreover, there was no significant difference between the control and vehicle groups in terms of MDA levels ( *p* >0.05). There is an antioxidant enzyme (SOD, CAT, and GPx) defence against this oxidative stress in the ovary. SOD, CAT, and GPx are antioxidant enzymes that play critical roles in converting radicals into nonradical products in the antioxidant defence mechanism ( 43 ). Melatonin increases the levels of antioxidant enzymes in different animal tissues ( 44 , 45 ). Mondal et al. (2017) reported that melatonin administration caused a significant decrease in MDA and an increase in SOD, CAT, GPx, GST, and GSH levels in the ovary in each reproductive phase. Consequently, these authors emphasized that the level of melatonin in the ovary was negatively correlated with MDA and positively correlated with SOD, CAT, and GPx levels ( 46 ). In another study, melatonin treatment led to a reduction in MDA levels in the ovary ( 47 ). Our results are also in line with those of previous studies. Melatonin is hypothesized to actively decrease oxidative stress and may also protect the ovaries against oxidative damage by elevating antioxidant enzyme activities.

The effect of melatonin on the ovary provides some benefits to the follicles through various mechanisms ( 14 , 48 ). The results of our study established that melatonin treatment induces an approximate two-fold increase in the number of healthy follicles. In contrast, the number of degenerated follicles significantly decreased with the application of melatonin treatment. These results indicate that melatonin treatment has a protective effect on follicles in the ovary in accordance with the literature.

In the current study, in response to melatonin treatment, the immunoreactivity of VEGF and VEGFR1 in rat ovaries paralleled the activity of antioxidant enzymes and lipid peroxidation.

We highlighted the effect of melatonin on the immunoreactivity of VEGF and its receptors in the ovary and critical regulatory roles in angiogenesis in physiological conditions. Further exploring the underlying mechanisms is necessary because the effects of melatonin on the VEGF, VEGFR1, and VEGFR2 pathways, which are intimately related to ovarian angiogenesis, have not completely been clarified.
